# Seroprevalence of pathogenic *Leptospira* serogroups in asymptomatic domestic dogs and cats: systematic review and meta-analysis

**DOI:** 10.3389/fvets.2024.1301959

**Published:** 2024-02-16

**Authors:** Tamara Ricardo, Lucía Isabel Azócar-Aedo, María Andrea Previtali, Gustavo Monti

**Affiliations:** ^1^Consejo Nacional de Investigaciones Científicas y Técnicas (CONICET), Santa Fe, Argentina; ^2^Dpto. de Ciencias Naturales, Facultad de Humanidades y Ciencias (FHUC), Universidad Nacional del Litoral (UNL), Santa Fe, Argentina; ^3^Escuela de Medicina Veterinaria, Facultad de Ciencias de la Naturaleza, Universidad San Sebastián, Sede de la Patagonia, Puerto Montt, Chile; ^4^Quantitative Veterinary Epidemiology Group, Wageningen University and Research, Wageningen, Netherlands

**Keywords:** leptospirosis, *Canis lupus familiaris*, *Felis silvestris catus*, pathogenic serotypes, meta-analysis

## Abstract

Leptospirosis is a neglected zoonotic disease transmitted by contact with the urine of animals infected with pathogenic species of the bacteria *Leptospira* or by contact with environments contaminated with the bacteria. Domestic dogs and cats may act as reservoirs or as sentinels of environmental contamination with leptospires, posing a public health concern. There is a great diversity of leptospires, and one common way to classify them is into serogroups that provide some information on the host species they are associated with. The aims of this study were: (1) to quantitatively summarize the overall prevalence and serogroup-specific prevalence of antibodies against pathogenic leptospires in asymptomatic dogs and cats and (2) to identify environmental and host characteristics that may affect the prevalence. Three electronic databases and the reference lists of eligible articles were screened, for epidemiological studies conducted between the years 2012–2022. We estimated overall and serogroup-specific prevalence using three-level meta-analysis models and assessed potential sources of heterogeneity by moderator analysis and meta-regression. Eighty-four studies met the inclusion criteria (dog studies 66.7%, cat studies 26.2%, and both species 7.1%). There were significant differences between dogs and cats in the overall prevalence model (*P* < 0.001), but not in the serogroup-specific model (*P*>0.05). In dogs, the prevalence of *Leptospira interrogans* serogroup Canicola was significantly higher than the other pathogenic serogroups (*P* < 0.001), while in cats there were no significant differences among serogroups (*P* = 0.373). Moderator analysis showed that the prevalence of *L. kirschneri* serogroup Grippotyphosa was significantly higher in stray/sheltered dogs than in domiciled dogs (*P* = 0.028). These results suggest that pathogenic serogroups associated with small mammals are circulating among asymptomatic pets and should be taken into account in the transmission cycle of leptospires, as well as in the standard MAT panel for diagnosis in dogs and cats. It also highlights the importance of including both dogs and cats as potential reservoirs when conducting eco-epidemiological studies in different geographical and ecological areas.

## 1 Introduction

Emerging and re-emerging infectious diseases are mainly of zoonotic origin and have a major impact on public health and the global economy (1, 2). Leptospirosis is a neglected zoonosis caused by spirochetes of the genus *Leptospira*, which have a complex transmission cycle at the ecosystem interface between animals, humans, and the environment (2). Human infections occur through direct contact with urine from infectious animals or indirectly by exposure to soil or water contaminated by the urine of infected animals (3, 4). Pathogenic *Leptospir*a spp. are generally endemic to certain regions and maintained by specific mammalian hosts, but can infect almost any animal species (3, 5, 6). These incidental infections are usually associated with acute clinical disease and limited renal excretion, whereas maintenance hosts often have subclinical infections and may shed leptospires in their urine for prolonged periods (3, 5). Given the complex transmission process, an effective and sustainable prevention and control strategy requires the application of the “One Health” approach (1, 2). This approach is particularly relevant to epidemiological studies in companion animals because of the close relationship between dogs, cats, and humans, the main route of transmission being urine-contaminated soil or water, and the influence of environmental conditions on bacterial survival, increasing the risk of shared exposure to pathogenic *Leptospira* spp. (3, 7).

Domestic dogs (*Canis lupus familiaris*) have traditionally been considered a maintenance host for *Leptospira interrogans* serovar Canicola, but there is evidence of severe disease in dogs infected with this serovar and reports of chronic infection and possible renal shedding of other commonly detected serovars (3, 5). Although the main clinical signs of leptospirosis in dogs reflect acute tubulointerstitial nephritis and liver dysfunction, the disease is multisystemic, with clinical signs of respiratory, intestinal, muscular, ocular, and reproductive problems, as well as coagulopathies (3). The risk of exposure to pathogenic leptospires in dogs is increased by unsupervised access to the outdoors, poor hygiene, and behaviors such as scavenging for litter or sniffing and licking urine from other dogs (3, 8). Despite this, further research on the serovars associated with both clinical and subclinical infections is still necessary.

Domestic cats (*Felis silvestris catus*) are also environmentally exposed to pathogenic leptospires due to their rodent hunting habits and, in many areas of the world, by their free-roaming lifestyle (9–12). Because cats are less likely to present clinical signs than dogs (3, 13), fewer studies have been conducted to investigate the clinical manifestations associated with all the serovars reported in this species or to understand the epidemiology of infection. However, some studies reported the presence of anorexia, dyspnea, chronic diarrhea, interstitial nephritis, and hepatitis in cats infected with pathogenic *Leptospira* spp. (14–18). The International Society of Companion Animal Infectious Diseases (ISCAID) statement indicated that the main serovars detected in this species are *L. interrogans* serovars Icterohaemorrhagiae, Canicola, Pomona, Bratislava, and Autumnalis; *L. kirschneri* serovar Grippotyphosa; and *L. borgpetersenii* serovars Hardjo and Ballum (6). However, there are still controversies on whether they are maintenance hosts or incidental hosts (3, 12, 19). In addition, recent epidemiological studies detected the DNA of pathogenic leptospires in the kidney and urine of domestic cats, indicating a potential role in environmental transmission (10, 20–25).

The care of companion animals and the closeness of human–pet relationships can increase the risk of zoonotic disease transmission (7, 26). For this reason, epidemiologic studies aimed at determining the frequency of presentation and prevalence of zoonoses in animal populations are of great relevance for detecting human and animal populations at risk (27) or differentiating risk areas for control purposes (28–30). Direct transmission of leptospires from dogs or cats to humans is a subject of controversy (3, 6, 12, 19). However, both species may act by zoonotic spillover from rodents or livestock to humans, as sentinels of environmental contamination with pathogenic leptospires, or contribute to contamination of the surroundings of human dwellings (3, 4); thus, there is a need to generate more evidence on this potential risk.

As the immunity induced by vaccination with current *Leptospira* bacterins is serogroup specific, it is important to recognize the serogroups that commonly cause disease in a given geographical region when designing a new vaccine or updating an existing one (3, 31, 32). Commercial leptospirosis vaccines for companion animals are currently available for dogs but not for cats (3, 6, 19). These vaccines generally contain a combination of *L. interrogans* serovars Canicola and Icterohaemorrhagiae, but the emergence of canine leptospirosis caused by other serovars has led to the development of multivalent vaccines containing *L. interrogans* serovars Pomona, Bratislava or Australis, and/or *L. kirschneri* serovar Grippotyphosa (3, 32). Furthermore, they are thought to provide protection for < 12 months (12, 33) and it is not clear whether commercial vaccines provide cross-protection against other serogroups not included in the formulation of inactivated bacterins (32). It is worth noting that animals vaccinated can present a reaction to serological tests even if they are not infected, which would interfere in serological surveys, for example, by overestimating the prevalence rate, or when validating serological tests affecting sensitivity and specificity estimations.

The microagglutination test (MAT) is the most widely used diagnostic technique for leptospirosis in veterinary practice. However, it has limitations in terms of sensitivity and specificity (3, 31). Several studies have used MAT to investigate asymptomatic infections in domestic dogs and cats, but there is no consensus on the most appropriate MAT titer, the minimum number of serovars and which serovars should be included in the panel, and how to interpret samples that react to more than one serovar. As a result, comparisons of apparent prevalence between studies should be made with caution. This study was motivated by trying to shed light on the numerous gaps in the epidemiology of feline and canine leptospirosis, despite the publication of numerous studies. Therefore, the aims of this study were: (a) to quantitatively summarize the overall prevalence and serogroup-specific prevalence of antibodies against pathogenic *Leptospira* in asymptomatic dogs and cats and (b) to identify environmental and host characteristics that may affect the seroprevalence.

## 2 Materials and methods

This meta-analysis was conducted following the Preferred Reporting Items for Systematic Reviews and Meta-Analyses (PRISMA) standards (34). The study protocol was registered into the PROSPERO International Prospective Register of Systematic Reviews with the code CRD4202230129.

### 2.1 Inclusion and exclusion criteria

Eligible reports were cross-sectional and cohort studies that reported the presence of antibodies against pathogenic *Leptospira* in domestic dogs (*Canis lupus familiaris*) or cats (*Felis silvestris catus*) and were published in peer-reviewed journals between January 2012 and December 2022. Publications written in English, Spanish, Portuguese, French, Italian, and German language were considered eligible. The studies had to use MAT to determine the presence of leptospiral antibodies in apparently healthy dogs and cats of both sexes and of all ages, whether they were house pets or stray animals, regardless of their vaccination status. Publications with null seroprevalence, articles where collection of samples was performed before 2010, and those that included animals with comorbidities or clinical suspicion of leptospirosis were excluded. In addition, conference abstracts, systematic reviews, gray literature, non-peer-reviewed publications, experimental research, case–control studies, ecological studies, case reports, and case series were also excluded. Later in the evaluation process, eligible studies were excluded if the complete text or pertinent data, such as the number of samples reacting to more than one serovar and their titers, were unavailable, and not made available after contacting the authors.

### 2.2 Search strategy

The electronic databases PubMed, Scopus, and Dimensions were used to search for eligible studies. The search strategy was based on the components: “*Leptospira*,” “leptospirosis,” “dogs,” “canine,” “cats,” “feline,” “antibodies,” “prevalence,” “seropositivity,” and “infection” and was customized according to the characteristics of each database (Table 1). A “snowball search” was carried out to identify additional studies from the reference lists of eligible publications and systematic reviews on leptospiral infection in domestic dogs or cats.

**Table 1:** Search strings and date limits used in the database literature search.

**Database**	**Search string**	**Records**
PubMed	(((leptospira[Title/Abstract] OR leptospirosis[Title/Abstract]) AND (dogs[Title/Abstract] OR canine[Title/Abstract] OR cats[Title/Abstract] OR feline[Title/Abstract]) AND (antibodies[Title/Abstract] OR prevalence[Title/Abstract] OR seropositivity[Title/Abstract] OR infection[Title/Abstract])) AND ((“2012/01/01”[Date - Publication] : “2022/12/31”[Date - Publication]))) AND (“journal article”[Publication Type])	278
Scopus	TITLE-ABS-KEY ((leptospira OR leptospirosis) AND (dogs OR canine OR cats OR feline) AND (antibodies OR prevalence OR seropositivity OR infection)) AND PUBYEAR > 2011 AND PUBYEAR < 2023 AND (LIMIT-TO(EXACTKEYWORD, “Leptospirosis”) OR LIMIT-TO(EXACTKEYWORD, “Leptospira”)) AND (LIMIT-TO(DOCTYPE , “ar”)) AND (LIMIT-TO(SRCTYPE , “j”))	443
Dimensions	(leptospira OR leptospirosis) AND (dogs OR canine OR cats OR feline) AND (antibodies OR prevalence OR seropositivity OR infection); Publication Year: 2012–2022; Publication type: Article; Fields of Research (ANZSRC 2020): 3009 Veterinary Sciences	332
Total	—	1, 053

### 2.3 Study selection

Two review authors (TR and LAA) independently screened the titles and abstracts of the studies for eligibility, resolving any disagreement by consensus. Search results were screened and cleaned of duplicates using Mendeley Desktop^®^ software; duplicates that were not automatically detected were removed manually.

### 2.4 Data extraction

Data were collected collaboratively using a Google Sheets^®^ spreadsheet by the same two review authors who screened for eligible articles (TR and LAA). The outcomes considered as response variables were as follows: (1) the overall seroprevalence of leptospiral antibodies, estimated as the number of seropositive samples over the total number of samples and (2) the serogroup-specific seroprevalence, estimated as the number of seropositive samples for a pathogenic serogroup (including one or more frequent serovars) over the total number of samples. The data extraction spreadsheet included identifying variables (first author, title, journal, volume issue, ISSN, DOI, PMID), year of publication, the language of publication, year(s) of sampling, country of study, environmental setting (urban/peri-urban and rural), animals sampled (dogs and cats), the origin of animals (domiciled, sheltered, stray, and working dogs), MAT cut-off titer, the number of sampled animals, and the number of positive individuals for the overall population and per each detected serovar. Data on vaccination against *Leptospira* and serovars included in the vaccines were also collected for dog studies. Each row of the spreadsheet represented an effect estimate (*k*), and when a study presented results for different countries, sampling years, environmental settings, origins, vaccination status, or serovars, they were considered as separate effect estimates.

Free-roaming, unowned, dogs and cats were considered strays, regardless of their level of socialization with humans (20). Sheltered animals are stray dogs/cats housed in municipal or private shelters, which usually present overcrowding, poor hygienic conditions, and lack of veterinary care, increasing susceptibility to infections (35–37). Working dogs included those used by hunters to track feral pigs (*Sus scrofa*) and other wildlife, and dogs trained for herding, protecting properties, rescue operations, detecting drugs or explosives, or assisting people with disabilities (38–40). Each country was assigned to a geographic region and subregion based on the United Nations (UN) classification (https://unstats.un.org/unsd/methodology/m49/overview/). Information on the serovars used as antigens in the MAT panel, the number of serovars tested, the presence of coagglutinations or cross-reactions, and the individual risk-of-bias assessments were collected in a secondary spreadsheet.

### 2.5 Risk-of-bias assessment

Risk of bias (RoB) was assessed based on certain components of the STROBE-Vet statement (41). Each report was assessed independently by two reviewers (TR and LAA), with discrepancies resolved by consensus. Detailed information on the considered components can be found in Table 2. The overall RoB of each evaluation was calculated as the mean of the seven components and expressed as a percentage. The summary RoB was calculated as the mean of the overall RoB estimations from each reviewer. Scores ≤ 25% were considered high RoB, between 26% and 74% were considered medium RoB, and ≥75% were considered low RoB.

**Table 2:** Risk-of-bias (RoB) assessment tool for full-text articles based on seven components of the STROBE-Vet criteria (29).

**Component**	**Criterion**	**Score**
Study design	The study design is observational	0: not specified or incongruent; 0.5: can be inferred from the text; 1: detailed in the Methodology section
Objectives	The objective(s) of the study is/are stated	0: absent or inconsistent with results; 0.5: can be inferred from the text; 1: clearly stated in the text
Setting and dates	The geographic location in which the study was carried out and relevant dates, including periods of recruitment and data collection, are described	0: missing or non-described; 0.5: only the setting or dates were specified; 1: both items were clearly described
Eligibility criteria	The document indicates the eligibility criteria for the caretakers/managers and the animals, the sources, the methods of selection for the caretakers and the animals, and the method of follow-up (if it is applicable)	0: no eligibility criteria; 0.5: can be inferred from the text; 1: detailed in the Methodology section
Diagnostic criteria	The study clearly defines the diagnostic tests used and the diagnostic criteria	0: incomplete diagnostic criteria; 0.5: missing MAT panel or cut-off titer; 1: detailed in the Methodology section
Sample size	The document defines the sample size and how it was obtained	0: missing; 0.5: defined in the text; 1: detailed in the Methodology or Results section
Results	The main results are described (prevalence), or the number of positive animals, or unadjusted estimates and their precision [e.g., 95% Confidence Interval (CI)]	0: missing or incomplete; 0.5: missing relevant data; 1: data available in the main text or supplementary information

### 2.6 Statistical analysis

The individual estimations of seroprevalence were log-transformed, and a continuity correction of 0.5 was used for events with probabilities of 0 or 1. When a study reported that serum samples agglutinated for more than one serovar, the serovar with the highest titer was considered responsible for the infection (28, 42). If MAT titers were not available or the sample reacted with equal titers to more than one serovar, they were considered coagglutinations and excluded from the serogroup-level analysis. Effect estimates reporting the prevalence of vaccine serovars in dogs with up-to-date vaccination were excluded, regardless of the cut-off titer used and the percentage of vaccinated dogs in the sample.

The frequencies of categorical variables were compared using either Pearson's chi-square test or Fisher's exact test, and the frequencies of numerical variables were compared using either ANOVA or Kruskal–Wallis ANOVA tests (43, 44). To account for the lack of independence among effect estimates from the same study, we fitted three-level random-effect meta-analysis models. These models allow the estimation of three sources of variance: variance from effect estimates, within-study heterogeneity ($I_{\text{level}2}^{2}$), and between-study heterogeneity (*I*^2^_level3_) (45). All the model parameters were calculated using restricted maximum likelihood (REML). Results of the meta-analyses were back transformed as proportions and their 95% CI.

Potential sources of heterogeneity in the overall seroprevalence model were evaluated by moderator analyses. The categorical variables considered were environmental setting, the origin of the animals, vaccination status, and MAT cut-off titer. The sampling year was also considered as a continuous moderator for a meta-regression model. For the serogroup-specific seroprevalence models, moderator analysis was conducted by type of animal tested (dogs and cats) and serogroup. In addition, moderator analysis by origin of the animals and environmental setting was performed for serogroups detected in at least 20 dog studies. In the case of infrequent factor levels, similar categories were grouped and those with at least five observations were included in the subgroup analyses. Geographic regions and subregions were not considered for the moderator analysis due to the over-representation of studies from Latin America and the Caribbean. The presence of publication bias was assessed using Begg's test and Egger's test (46, 47). We performed a sensitivity analysis using a leave-one-out influence analysis, excluding the effect estimates with higher RoB (48). For the estimation of publication bias and sensitivity analysis, the models were refitted using two-level random-effects meta-analysis models. All the meta-analyses were performed in R software (49). Statistical significance was set at *p*-values below 0.05.

## 3 Results

### 3.1 Overview of the selected studies

**Figure 1:**
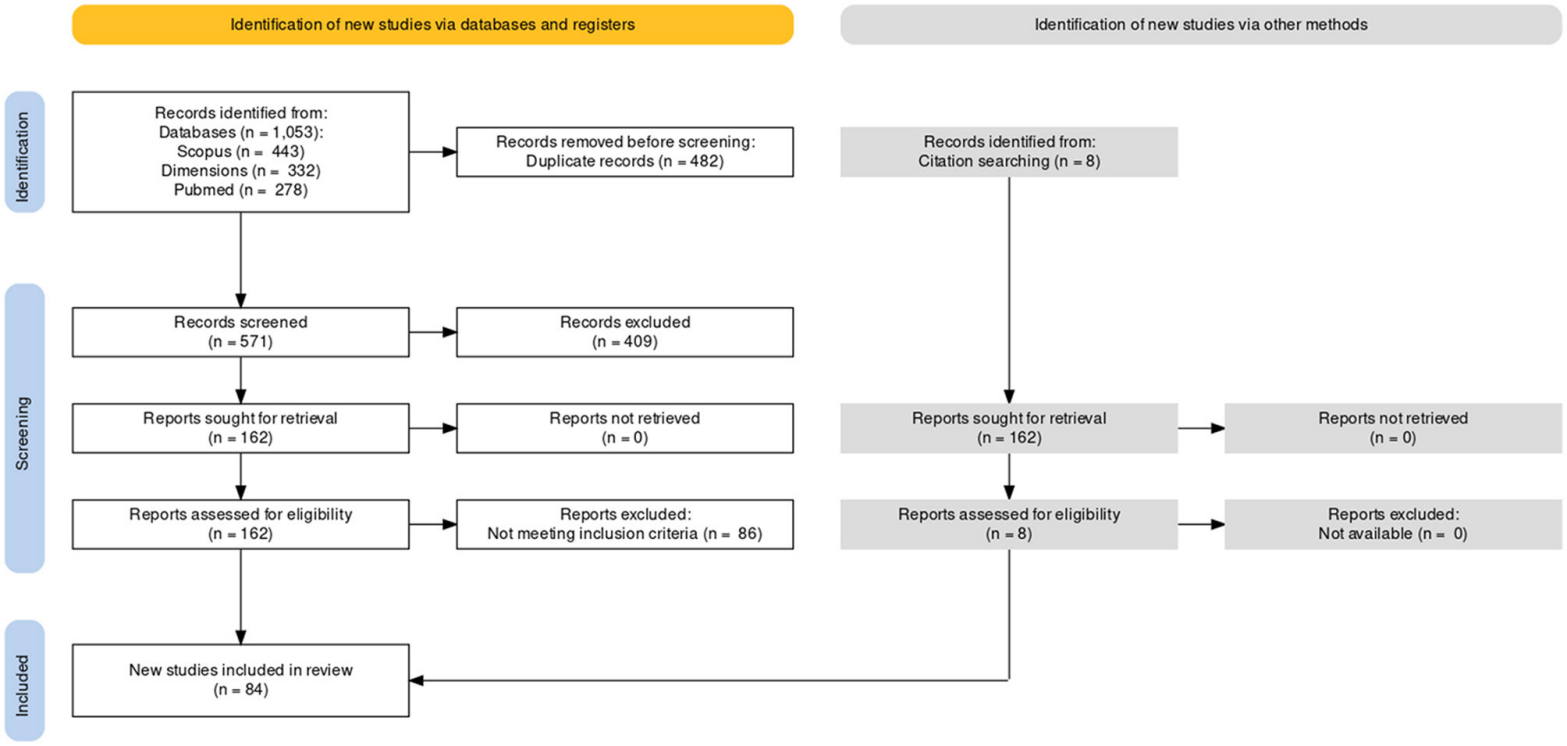
PRISMA flowchart for identification and selection of articles. Flow diagram generated using PRISMA2020: R package and ShinyApp for producing PRISMA 2020 compliant flow diagrams (Version 0.0.1) (https://www.eshackathon.org/software/PRISMA2020.html).

A literature search retrieved 1, 053 records, of which 162 were related to the presence of leptospiral antibodies in dogs or cats. Of these, we included 76 studies that met the inclusion criteria and 8 additional studies identified from the “snowball search” (Figure 1). Another 30 potentially eligible articles were excluded because the full text was not available (*n* = 3) or presented incomplete data (*n* = 27) and were not made available after contacting the correspondence authors. Based on the 84 selected studies, 66.7% sampled only dogs, 26.2% only cats, and 7.1% both species. The median estimation of risk of bias was 0.89 (IQR: 0.86; 0.96) and ranged between 0.61 and 1, which indicates that RoB was low in most of the studies (92.9%, Supplementary Table S1). The median for publication year was 2018 (IQR: 2016; 2020). The sampling year was missing in 14 studies (16.7%). No significant differences were observed regarding average RoB, publication year, or sampling year among studies that sampled dogs, cats, or both species (*P* > 0.05). The selected studies were performed in 28 different countries and territories, 11 geographic subregions, and 5 geographic regions. More than half of the studies (56.0%) were conducted in Latin America and the Caribbean, 20.2% in Southern/Southeastern Asia, 8.3% in Southern Europe, 4.8% in Northern America, 3.6% in Sub-Saharan Africa, and 2.4% each in Eastern Asia, Northern/Eastern Europe, and Oceania. The countries with the highest number of publications were Brazil (92.9%), Malaysia (9.5%), and Mexico (7.1%).

### 3.2 Prevalence of antibodies against *Leptospira* in asymptomatic dogs and cats

**Figure 2:**
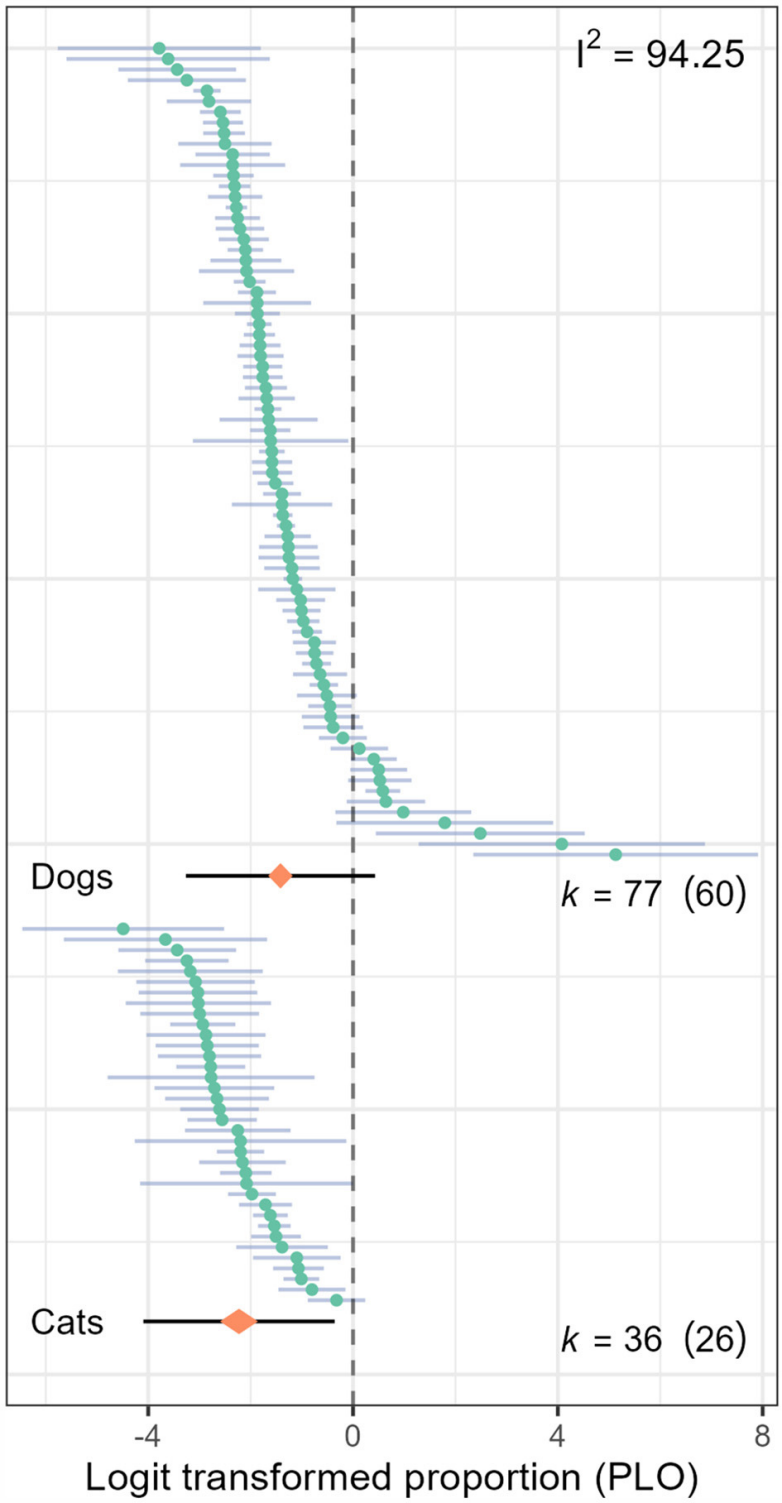
Caterpillar plot of the effect estimates (*k*) included in the overall seroprevalence model for leptospiral infection in dogs and cats. The orange diamond represents the overall estimator and its 95% confidence interval (CI); the black line represents the prediction interval, and the branches represent the individual effect sizes and their 95% CI.

We identified 77 effect estimates of seroprevalence in dogs (k _dogs_) and 36 effect estimates of seroprevalence in cats (k _cats_). The overall estimation of seroprevalence of *Leptospira* was 19.5% in dogs (95% CI: 16.0%; 23.5%, Figure 2) and 9.7% in cats (95% CI: 6.9%; 13.5%, Figure 2). Differences in the seroprevalence between dog studies and cat studies were statistically significant (*P* < 0.001). The model presented high statistical heterogeneity (I^2^: 94.2 %), and most of the variations corresponded to between-study heterogeneity ($I_{\text{level}3}^{2}$ = 60.8%, $I_{\text{level}2}^{2}$ = 33.4%). The authors did not detect evidence of publication bias in the dog model (*P*_*Begg*_ = 0.291, *P*_*Egger*_ = 0.29), but Egger's test showed evidence of publication bias in the cat model (*P*_*Begg*_ = 0.108, *P*_*Egger*_ < 0.001). Sensitivity analysis did not find significant differences after removing the studies with higher RoB from the dog model (21.2%, 95% CI: 16.8%; 26.5%) or the cat model (9.1%, 95% CI: 6.9%; 12.0%).

**Figure 3:**
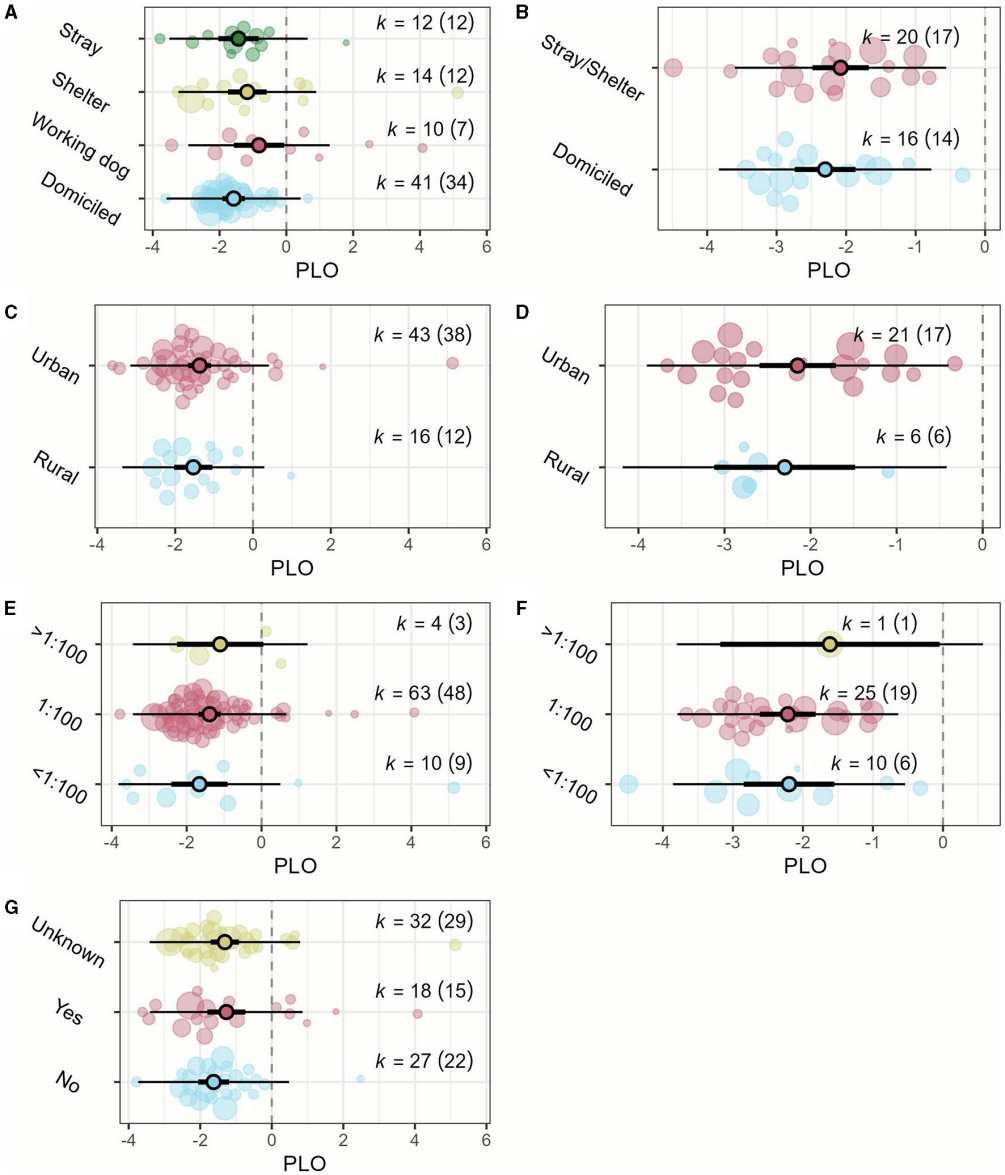
Orchard plots of the overall seroprevalence moderator analyses. **(A)** Origin of the animal (dogs); **(B)** origin of the animal (cats); **(C)** environmental setting (dogs); **(D)** environmental setting (cats); **(E)** MAT cut-off titer (dogs); **(F)** MAT cut-off titer (cats); **(G)** vaccination status (dogs). The central circles represent the mean effect size estimates, the bold branch represents its 95% confidence interval (CI), and the thin branches represent the prediction interval. Individual effect sizes, weighed by the study sample size, are represented as bubbles. PLO: logit transformed proportion.

Half of the effect estimates (50.4%) corresponded to domiciled animals (k_dogs_ = 41, k_cats_ = 16), 22.1% to strays (k_dogs_ = 12, k_cats_ = 13), 18.6% to sheltered (k_dogs_ = 14, k_cats_ = 7), and 8.8% to working dogs (k_dogs_ = 10). Most of the effect estimates (56.6%) were from urban areas (k_dogs_ = 43, k_cats_ = 21), 19.5% from rural areas (k_dogs_ = 16, k_cats_ = 6), while 23.9% did not specify the living environment of the sampled animals. The MAT cut-off titer of 1:100 was used in 77.9% of effect estimates (k_dogs_ = 16, k_cats_ = 6), 17.7% used titers below 1:100 (k_dogs_ = 43, k_cats_ = 21), and 4.4% used titers above 1:100 (k_dogs_ = 18, k_cats_ = 9). The vaccination status of dogs was uncertain in 41.6% of the effect estimates. Among the known cases, 23.4% involved dogs with up-to-date leptospirosis vaccination, while 35.1% were unvaccinated dogs. Among the 18 effect estimates from vaccinated dogs, 50.0% used a bivalent vaccine containing *L. interrogans* serovars Icterohaemorrhagiae and Canicola, 16.7% used a tetravalent vaccine containing *L. interrogans* serovars Icterohaemorrhagiae, Canicola, and Pomona and *L. kirschneri* serovar Grippotyphosa; 11.1% used a tetravalent vaccine containing *L. interrogans* serovars Icterohaemorrhagiae, Canicola, and Australis and *L. kirschneri* serovar Grippotyphosa; and the remaining 23.5% used other combinations of serovars. The results of the moderator analysis showed that there was not a significant moderating effect of the origin of the animals (*P*_*dogs*_ = 0.277, *P*_*cats*_ = 0.414, Figure 3), their living environment (*P*_*dogs*_ = 0.56, and *P*_*cats*_ = 0.73, Figure 3), MAT cut-off titer used (*P*_*dogs*_ = 0.693, and *P*_*cats*_ = 0.751, Figure 3), and vaccination status of dogs (*P*_*dogs*_ = 0.392, Figure 3). The moderator analysis also did not detect a significant time trend in either dog studies or cat studies (*P*_*dogs*_ = 0.189, and *P*_*cats*_ = 0.689, Figure 3).

### 3.3 Serogroup and serovar-specific seroprevalence

The selected studies tested a total of 74 serovars belonging to 26 known serogroups, plus two serovars of undetermined serogroups (Cantagalo and Khorat, Supplementary Tables S1, S2). The median number of serovars tested per study was 14 (IQR: 9–22), with a minimum of two and a maximum of 27 (Supplementary Tables S1, S2). Four studies had incomplete information on the serovars used in the MAT panel (Supplementary Table S1). Among the 76 tested serovars, 50 (67.6%) were detected in the sampled animals, of which 22 (44%) were detected in both species, 25 (50%) only in dogs, and *L. biflexa* serovar Andamana, *L. borgpetersenii* serovar Arborea, and *L. interrogans* serovar Rachmati only in cats (Supplementary Table S2).

**Figure 4:**
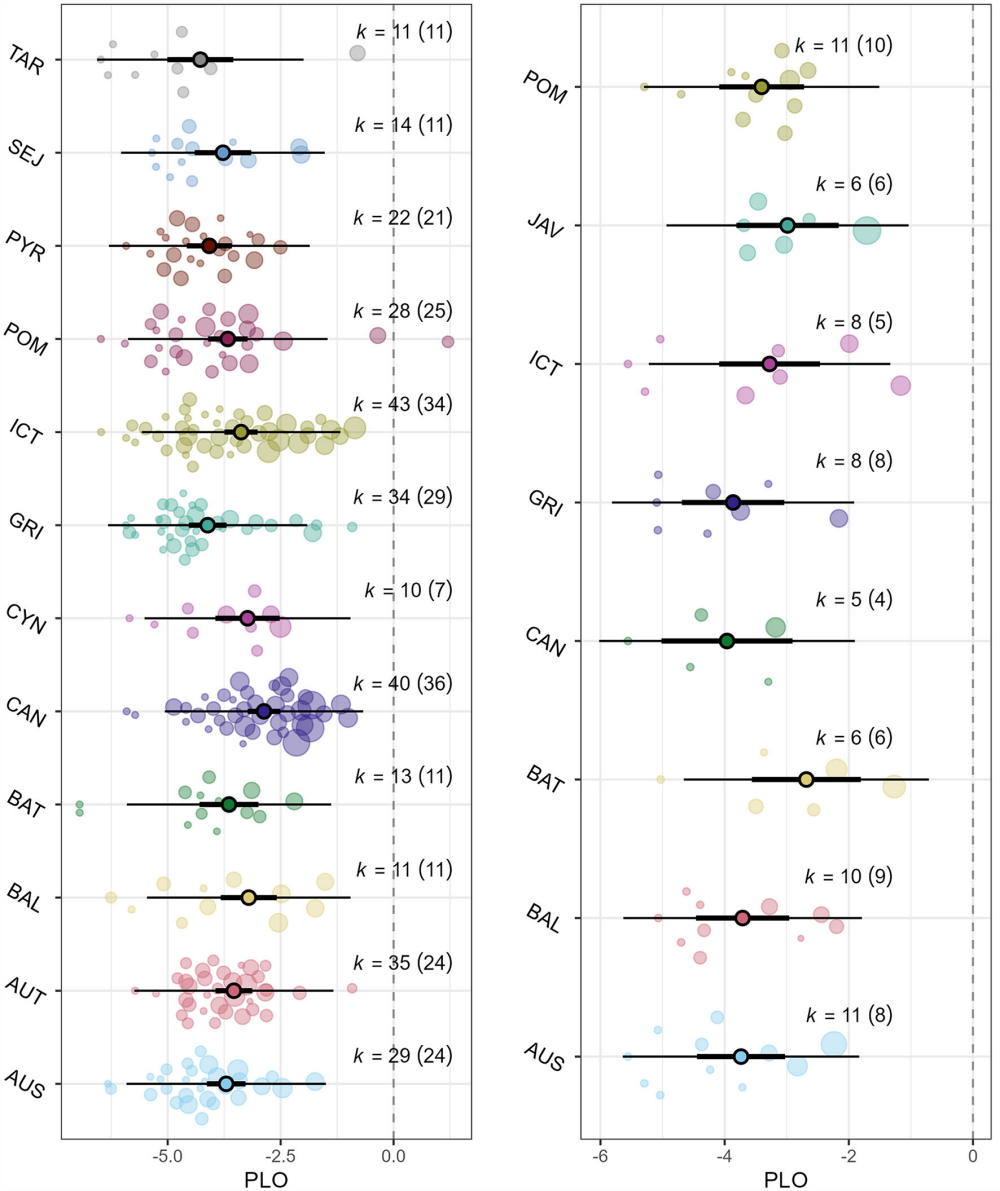
Orchard plots of the serogroup-specific seroprevalence analyses. **(A)** Dog studies; **(B)** cat studies. The central circles represent the mean effect size estimates, the bold branch represents its 95% confidence interval (CI), and the thin branches represent the prediction interval. Individual effect sizes are represented as bubbles. PLO: logit transformed proportion.

We identified 17 pathogenic serovars within 13 serogroups that appeared in at least 10 effect estimates from dogs and/or five effect estimates from cats. The overall estimation of seroprevalence for any of these pathogenic serogroups was 2.8% (95% CI: 2.3%; 3.4%), with no significant differences between dog studies and cat studies (*P* = 0.516) and high statistical heterogeneity (I^2^ = 85.8%). Based on 290 effect estimates from dog studies, there were significant differences in the seroprevalence of the 12 most frequent pathogenic serogroups (*P* < 0.001), with *L. interrogans* serogroup Canicola presenting the highest seroprevalence (5.4%, 95% CI: 3.8%; 7.5%), and *L. interrogans* serovar Pyrogenes (1.7%, 95% CI: 1.0%; 2.7%), *L. kirschneri* serogroup Grippotyphosa (1.6%, 95% CI: 1.1%; 2.4%), and *L. borgpetersenii* serovar Tarassovi (1.4%, 95% CI: 0.7%; 2.8%) the lowest (Figure 4). No significant differences were detected in the seroprevalence of the eight most frequent pathogenic serogroups in cat studies (*P* = 0.373, Figure 4).

For dog studies, we fitted individual meta-analysis models to evaluate variables that could affect the seroprevalence in serogroups with at least 20 effect estimates. Seroprevalence of *L. kirschneri* serogroup Grippotyphosa was significantly higher (*P* = 0.028) in stray and sheltered dogs (2.8%, 95% CI: 1.4%;5.5%) than domiciled dogs (1.1%, 95% CI: 0.7%;1.8%). No other significant differences were observed regarding the origin or environmental setting of the dogs (*P* > 0.05). Subgroup analyses for cat studies were not performed due to the small number of effect estimates.

## 4 Discussion

In this systematic review and meta-analysis, we estimated the seroprevalence of the most frequently detected pathogenic serogroups in asymptomatic dogs and cats in the period 2012–2021, as well as those with greater prevalence in dogs (*L. interrogans* serogroups Canicola and Autumnalis, *L. borgpetersenii* serovar Ballum), cats (*L. borgpetersenii* serovar Javanica), and both species (*L. interrogans* serogroups Icterohaemorrhagiae, Bataviae, and Pomona). Previous systematic reviews had reported that *L. interrogans* serogroups Australis, Canicola, Icterohaemorrhagiae, and Pomona; *L. kirschneri* serogroup Grippotyphosa; and *L. borgpetersenii* serogroup Sejroe were the most prevalent in dogs (5, 31), and *L. interrogans* serogroups Autumnalis, Canicola, Icterohaemorrhagiae, and Pomona; *L. kirschneri* serogroup Grippotyphosa; and *L. borgpetersenii* serogroup Ballum were the most prevalent in cats (6, 19). Most of these reviews, however, covered different time frames and were limited to Northern Hemisphere countries, and the prevalence of a pathogenic serogroup may present spatiotemporal variations (4, 50). It should also be noted that in our study, the seroprevalence of a particular serogroup was estimated by dividing the number of seropositive individuals by the sample size, and in case of agglutination to multiple serovars, only the one with the highest titer was accounted, which may have lowered the pooled estimates.

In our study, the largest estimation of seroprevalence for domestic dogs corresponded to *L. interrogans* serogroup Canicola. Dogs are the only known maintenance hosts of this serogroup, and exposure is thought to occur mainly through contact with the urine of infected dogs (5, 31). Moreover, the estimated seroprevalence of *L. interrogans* serogroup Canicola in domestic cats was low (1.9%), which is consistent with published studies that reported that the risk of transmission from dogs to cats seems to be low (6, 51, 52). However, recent evidence suggests that rodents, horses, and pigs may also play a significant role in the environmental transmission cycle of this serovar (3).

In our study, *L. interrogans* serogroup Icterohaemorrhagiae was a serogroup frequently found in both species. Rats (*Rattus* spp.) are the major maintenance hosts for this serogroup (12, 31). Infection in dogs is thought to occur mainly through contact with rat urine and it is usually associated with severe forms of leptospirosis (6, 33), whereas in cats, infections with this serogroup may occur by rodent hunting and there are no reports of clinical illness (6, 12). However, there is evidence of renal excretion in asymptomatic dogs and cats seropositive to *L. interrogans* serogroup Icterohaemorrhagiae (15, 20, 21, 53, 54). The detection of this serogroup in asymptomatic pets should be of public health concern, as infections with Icterohaemorrhagiae in humans are associated with severe forms of leptospirosis, and domestic dogs and cats may be acting as epidemiological links between infected rats and pet caretakers (55, 56). These results can be related to the One Health concept as they illustrate the links between environmental, animal, and human health. From this perspective, a disturbed ecosystem harboring a large rat population could be contaminated with pathogenic leptospires, which could consequently affect human and animal health. Solutions to such a complex scenario can only be found through the joint efforts of all relevant disciplines and sectors (1, 3, 4).

The seroprevalence of *L. interrogans* serovars Canicola and Icterohaemorrhagiae in domestic dogs has shown a downward trend in the northern hemisphere over the past few decades, thought to be related to the widespread use of bivalent vaccines (5). This trend contrasts with the relatively high overall pooled frequencies of these serogroups estimated in our meta-analysis and Esteves et al. (31) and may be linked to the different epidemiological contexts found in countries from Latin America and the Caribbean, South and Southeast Asia, and the relatively lower adoption coverage of where canine vaccination is not mandatory. In these areas, the prices of the vaccines are not quite affordable for a large proportion of pet caretakers, and many of them are still unaware of the importance of vaccinating their pets regularly, especially with vaccines other than rabies (31, 57).

*L. interrogans* serogroup Pomona is maintained by pigs and cattle and was found in the kidneys of skunks and opossums (6, 12). Our results show that this serogroup is quite common in both species, dogs and cats, and its prevalence did not differ significantly between animals living in urban or rural environments, which is in contrast to the findings of Esteves et al. (31). It should be noted that many studies from urban areas have been conducted in slums or peri-urban settlements, where residents tend to keep subsistence livestock animals in backyards without veterinary care and exposed to garbage and wildlife, increasing the likelihood of transmission of leptospires to companion animals (58, 59). It should also be noted that most of the studies that included working dogs, involved hunting dogs, which are in close contact with feral pigs and small mammals and may act as epidemiological links of infections with serogroup Pomona as well (60, 61).

*L. interrogans* serogroups Autumnalis and Bataviae and *L. borgpetersenii* serogroup Javanica are also associated with wild rodents, rats (*Rattus* spp.), and small mammals (62–65), whereas the main reservoirs of *L. borgpetersenii* serogroup Ballum are house mice (*Mus musculus)* (6, 66). These serogroups were detected in several dog and cat studies, which may be indicative of direct exposure by rodent hunting, or indirectly by contact with garbage, chicken huts, and other areas contaminated with rodent urine (58, 67–69). However, *L. interrogans* serogroup Autumnalis presents cross-reactivity with *L. interrogans* serogroups Pomona, Canicola, and Icterohaemorrhagiae, and its large prevalence should be interpreted with caution (70, 71).

The results of this meta-analysis showed that there were no significant differences in the seroprevalence to a single pathogenic serogroup between dogs and cats (*P* = 0.648). This may be explained considering that it is rather frequent that the same animal reacts to more than one serovar, sometimes with the same titer, making it impossible to determine whether the animal is infected with multiple serovars or there were cross-reactions between antigenically related serovars (6, 71). To improve knowledge of the species of *Leptospira* infecting domestic dogs and cats and their maintenance hosts, future epidemiological studies should combine serological and molecular characterization (3, 8, 9, 31).

This study has some limitations worth noting. First, a large proportion of the studies included in the analysis were conducted in Brazil and may not be representative of the worldwide distribution and seroprevalence of pathogenic *Leptospira* in domestic dogs and cats, and it might influence the pooled estimates. However, publication bias assessment did not suggest any significant effect on that. Second, the number of cat studies found was relatively low, which prevented us from performing subgroup analyses and meta-regression for cats. Third, statistical heterogeneity was high, which can be attributed to the presence of confounding factors that were not considered in the base studies, as well as the over-representation of studies from Brazil. Finally, it should be noted that even though the MAT is specific to serogroup levels, cross-reactions still may occur among antigenically similar serogroups. It should be noted that vaccinated animals may react to serological tests even if they are not infected, which may affect prevalence estimates in serological surveys, especially for the serovars included in the vaccines used in those populations. Despite the lack of specificity of the MAT and other limitations related to the technique mentioned before, we consider that the results of this meta-analysis are of relevance for both veterinary and human health as they show a large burden of infection in a group of animal species with important bonds with humans.

We believe that the results of our study will be useful to health professionals and researchers not only to better understand the pathogenic serogroups of *Leptospira* currently circulating in asymptomatic companion animals, their potential maintenance hosts, and the risk of environmental contamination to other animal species and humans but also to promote adoption of biosecurity measures and appropriate handling precautions when humans handle or are exposed to companion animals. Our results would also be useful in the development of public health strategies aimed at reducing the transmission of pathogenic leptospires from the environment to domestic and stray animals and the potential risk of transmission to humans.

## Author contributions

TR: Data curation, Formal analysis, Investigation, Methodology, Visualization, Writing—original draft, Writing—review & editing. LA-A: Data curation, Investigation, Methodology, Writing—original draft, Writing—review & editing. MP: Resources, Supervision, Writing—original draft, Writing—review & editing. GM: Conceptualization, Investigation, Resources, Supervision, Writing—original draft, Writing—review & editing.
