# Supplementary material for: Seroprevalence of pathogenic Leptospira serogroups in asymptomatic domestic dogs and cats: systematic review and meta-analysis
Source: Front Vet Sci. 2024 Feb 16;11:1301959. doi: 10.3389/fvets.2024.1301959 (PMC10904519; doi:10.3389/fvets.2024.1301959)
Supplement: Supplementary file 1 [file Table_1.DOCX]

Supplementary material 1

**Seroprevalence of pathogenic *Leptospira* serogroups in asymptomatic dogs and cats: systematic review and meta-analysis**

Tamara Ricardo, Lucía Isabel Azócar-Aedo, María Andrea Previtali, Gustavo Monti*

*Correspondence: Gustavo Monti: [gustavo.monti@wur.nl](mailto:gustavo.monti@wur.nl)

## Supplementary Tables

Supplementary Table 1. Characteristics of the studies included in the meta-analysis. RoB: average risk of bias; SY: sampling year; N: number of serovars included in the MAT panel. Serovar names were abbreviated to the first three letters of their names, except for serovars Cantagalo (CANT), Hardjobovis (HARB), Manilae (MANI) and Minis (MINIS). N/S: not specified.

| AU | Animal | RoB | SY | Country | N | Serovars |
| --- | --- | --- | --- | --- | --- | --- |
| Alashraf et al. (2019) | Cats | 0.79 | 2017 | Malaysia | 20 | AUS, AUT, BAL, BAT, CAN, CEL, COP, CYN, DJA, GRI, HAR, HARB, HEB, ICT, JAV, LAI, MAL, POM, PYR, TAR |
| Alashraf et al. (2020a) | Cats | 0.79 | 2017 | Malaysia | 20 |  |
| Alashraf et al. (2020b) | Dogs and cats | 0.86 | 2017 | Malaysia | 21 | AUS, AUT, BAL, BAT, CAN, CEL, COP, CYN, DJA, GRI, HAR, HARB, HEB, ICT, JAV, LAI, MAL, PAT, POM, PYR, TAR |
| Albuquerque et al. (2020) | Dogs | 1.00 | 2018 | Brazil | 19 | AND, AUS, AUT, BAL, BAT, CAN, CEL, CYN, DJA, GRI, HEB, ICT, PAN, POM, PYR, SEJ, SEM, SHE, TAR |
| Arzamani et al. (2022) | Dogs | 0.82 | 2020 | Iran | 9 | AUS, AUT, BAL, CAN, GRI, HAR, ICT, POM, TAR |
| Athapattu et al. (2022) | Dogs | 0.93 |  | Sri Lanka | 13 | AUT, BAT, CAN, COP, GRI, HAR, HEB, ICT, JAV, PAN, PYR, SHE, TAR |
| Azócar-Aedo et al. (2014) | Cats | 0.96 | 2011 | Chile | 18 | AUS, AUT, BAL, BAT, CAN, CYN, GRI, HAR, HEB, ICT, JAV, PAN, PAT, POM, PYR, SEJ, TAR, WOL |
| Blum Domínguez et al. (2013) | Dogs | 0.96 |  | Mexico | 9 | BAT, CAN, GRI, HAR, ICT, POM, PYR, SEJ, TAR |
| Bourassi et al. (2021) | Cats | 0.93 | 2017 | Canada | 6 | BRA, CAN, GRI, HAR, ICT, POM |
| Caldart et al. (2015) | Dogs | 0.89 | 2010 | Brazil | 20 | AUS, AUT, BAT, BRA, BUT, CAN, CAS, COP, CYN, FOR, GRI, HAR, HEB, ICT, PAN, POM, PYR, SHE, WHI, WOL |
| Castrillón-Salazar et al. (2018) | Dogs | 0.89 | 2016 | Colombia | 8 | AUT, BAL, BRA, CAN, GRI, ICT, POM, TAR |
| Chan et al. (2014) | Cats | 0.89 | 2010 | Taiwan, China | 11 | AUS, AUT, BAT, CAN, ICT, JAV, PAN, POM, PYR, SHE, TAR |
| Cilia et al. (2021) | Dogs | 0.89 | 2020 | Italy | 8 | BAL, BRA, CAN, GRI, HAR, ICT, POM, TAR |
| Cordeiro et al. (2017) | Cats | 0.89 | 2016 | Brazil | 11 | AUT, BRA, CAN, CAS, COP, CYN, GRI, HAR, ICT, POM, PYR |
| Cruz-Romero et al. (2013) | Dogs | 0.89 |  | Mexico | 12 | AUT, BAL, BRA, CAN, GRI, HAR, ICT, LAI, MUE, POM, PYR, TAR |
| Cútuk et al. (2020) | Dogs | 0.96 | 2014 | Bosnia and Herzegovina | 12 | AUS, AUT, BAT, BRA, CAN, GRI, HAR, ICT, POM, SAX, SEJ, TAR |
| Donato et al. (2022) | Cats | 0.96 | 2018 | Italy | 14 | ARB, BAL, BRA, CAN, COP, GRI, HAR, ICT, LOR, MIN, PAT, POI, POM, TAR |
| Felix et al. (2020) | Dogs | 0.82 |  | Brazil | 2 | CAN, ICT |
| Fernandes et al. (2013) | Dogs | 0.96 | 2011 | Brazil | 24 | AUS, AUT, BAT, BRA, BUT, CAN, CAS, COP, CYN, GRI, HAR, HARB, HEB, ICT, JAV, PAN, PAT, POM, PYR, SEN, SHE, TAR, WHI, WOL |
| Fernandes et al. (2018a) | Dogs | 0.93 | 2014 | Brazil | 18 | AUS, AUT, BAT, BRA, CAN, CAS, COP, CYN, DJA, GRI, GUA, HAR, HEB, ICT, POM, SEJ, TAR, WOL |
| Fernandes et al. (2018b) | Dogs | 1.00 | 2013 | Brazil | 20 | AUS, AUT, BAT, BRA, CAN, CAS, COP, CYN, DJA, GRI, GUA, HAR, HARB, HEB, ICT, PAN, POM, SEJ, TAR, WOL |
| Francois et al (2020) | Cats | 0.75 | 2016 | Argentina | 11 | AUT, BAT, BRA, CAN, CAS, COP, CYN, GRI, HAR, POM, PYR |
| Gay et al. (2014) | Dogs | 0.86 | 2010 | New Caledonia | 12 | AUS, AUT, BAT, CAN, CAS, COP, ICT, PAN, PAT, POM, PYR, TAR |
| Gloriani et al. (2016) | Dogs | 0.71 |  | Philippines | 17 | AUS, AUT, CAN, COP, GRI, HAR, HEB, ICT, LOS, MANI, PAT, POI, POM, PYR, RAT, SEM, TAR |
| Goh et al. (2020) | Dogs | 0.75 | 2017 | Malaysia | 20 | AUS, AUT, BAL, BAT, CAN, CEL, COP, CYN, GRI, HAR, HARB, HEB, ICT, JAV, LAI, MAL, PAT, POM, PYR, TAR |
| Goh et al. (2021) | Dogs | 0.89 |  | Malaysia | 20 | AUS, AUT, BAL, BAT, CAN, CEL, COP, CYN, DJA, GRI, HARB, HEB, ICT, JAV, LAI, MAL, PAT, POM, PYR, TAR |
| Hafemann et al. (2018) | Dogs | 0.89 | 2015 | Brazil | 22 | AUS, AUT, BAT, BRA, BUT, CAN, CAS, COP, CYN, FOR, GRI, HAR, HEB, ICT, PAN, POM, PYR, SEN, SHE, TAR, WHI, WOL |
| Hernández Ramírez et al. (2017b) | Dogs | 0.75 |  | Mexico | 12 | AUS, BAL, BRA, CAN, GRI, HAR, HEB, ICT, POM, PYR, SHE, WOL |
| Holzapfel et al. (2021) | Cats | 0.96 | 2013 | Reunion Island | 25 | AUS, AUT, BAT, BIM, BRA, CAN, CAS, COP, CYN, GRI, HAR, ICT, KRE, MIN, MUE, PAN, PAT, POM, PYR, SAX, SEJ, SEM, TAR, VAN, WOL |
| Kakita et al. (2021) | Cats | 0.93 | 2012 | Japan | 13 | AUS, AUT, BAT, CAN, CAS, GRI, HAR, HEB, ICT, JAV, POM, PYR, RAC |
| Khomayezi et al. (2015) | Cats | 0.89 | 2011 | Iran | 8 | AUS, AUT, BAL, CAN, GRI, HAR, ICT, POM |
| Khor et al. (2016) | Dogs | 0.82 | 2014 | Malaysia | 10 | AND, AUS, BAT, CAN, GRI, HEB, ICT, LAI, POM, TAR |
| Latosinski et al. (2018) | Dogs | 0.79 | 2014 | Brazil | 11 | AUS, AUT, BRA, CAN, COP, GRI, HAR, ICT, POM, PYR, SEN |
| Lau et al. (2016) | Dogs | 0.79 | 2014 | Malaysia | 10 | AND, AUS, BAT, CAN, GRI, HEB, ICT, POM, SHE, TAR |
| Lau et al. (2017) | Dogs | 0.86 | 2015 | Malaysia | 11 | AUS, BAT, CAN, GRI, HEB, ICT, JAV, LAI, POM, PYR, TAR |
| Lelu et al. (2015) | Dogs | 0.86 | 2010 | Chile | 20 | ALE, AUS, AUT, BAL, BAT, BOR, BRA, CAN, CEL, CYN, DJA, GEO, GRI, ICT, JAV, MAN, POM, PYR, TAR, WOL |
| Lemos et al. (2020) | Dogs | 0.93 | 2017 | Brazil | 24 | AND, AUS, AUT, BAT, BRA, BUT, CAN, CAS, COP, CYN, GRI, HAR, HEB, ICT, JAV, PAN, PAT, POM, PYR, SEN, SHE, TAR, WHI, WOL |
| Loffler et al. (2016) | Dogs | 0.89 |  | Argentina | 10 | CAN, CAS, GRI, HAR, HEB, ICT, POM, PYR, TAR, WOL |
| Machado et al. (2021) | Dogs | 0.71 | 2016 | Brazil | 26 | AND, AUS, AUT, BAT, BRA, CAN, CAS, COP, CYN, DJA, GRI, GUA, HAR, HARB, HEB, ICT, JAV, PAN, PAT, POM, PYR, SEN, SHE, TAR, WHI, WOL |
| Mai et al. (2021) | Dogs and cats | 0.89 | 2019 | Viet Nam | 25 | AUS, AUT, BAT, BRA, CAN, CAS, CEL, COP, CYN, DJA, GRI, HARB, HEB, ICT, JAV, LOU, MIN, PAN, PAT, POM, PYR, SHE, TAR, VUG, WOL |
| Manić et al. (2015) | Dogs | 0.61 |  | Serbia | 8 | AUS, BAT, BRA, CAN, GRI, ICT, POM, SEJ |
| Markovich et al. (2012) | Cats | 0.89 | 2010 | United States | 7 | AUT, BRA, CAN, GRI, HAR, ICT, POM |
| Martins et al. (2013) | Dogs | 0.93 | 2010 | Brazil | 12 | AUS, AUT, BRA, CAN, COP, CYN, DJA, GRI, HAR, ICT, POM, PYR |
| Miotto et al. (2018a) | Dogs | 0.89 | 2013 | Brazil | 22 | AUS, AUT, BAT, BRA, BUT, CAN, CAS, COP, CYN, GRI, GUA, HAR, HEB, ICT, JAV, PAN, POM, PYR, SEN, SHE, TAR, WHI |
| Moraes et al. (2020) | Dogs | 0.93 | 2016 | Brazil | 24 | AND, AUS, AUT, BAT, BRA, BUT, CAN, CAS, COP, CYN, GRI, HAR, HEB, ICT, JAV, PAN, PAT, POM, PYR, SEN, SHE, TAR, WHI, WOL |
| Msemwa et al. (2021) | Dogs | 1.00 | 2018 | Tanzania | 5 | GRI, HEB, KEN, POM, SOK |
| Muliany et al. (2018) | Cats | 0.75 |  | Indonesia | 13 | AUS, BAL, BAT, CAN, CEL, CYN, GRI, HAR, ICT, JAV, PYR, RAC, TAR |
| Murcia et al. (2020) | Dogs | 1.00 | 2017 | Colombia | 24 | AUT, BAL, BAT, BRA, CAN, CEL, CYN, DJA, GRI, HAR, HEB, HUR, ICT, LIC, LOU, MIN, PAN, PAT, POM, PYR, RAN, SAR, SHE, TAR |
| Murillo et al. (2020) | Cats | 0.93 | 2017 | Spain | 27 | AND, AUS, BAL, BAT, BRA, CAN, CEL, COP, CYN, GRI, HAR, HARB, HEB, ICT, MIN, PAN, PAT, POI, POM, PRO, PYR, RAC, SAX, SEJ, SEM, SHE, TAR |
| Obrenovic et al. (2014) | Cats | 0.89 | 2012 | Serbia | 10 | AUS, AUT, BAT, BRA, CAN, GRI, ICT, POM, PYR, SEJ |
| Orr et al. (2022) | Dogs | 1.00 | 2018 | Australia | 22 | ARB, AUS, BAT, BUL, CAN, CEL, COP, CYN, DJA, GRI, HAR, JAV, KRE, MED, PAN, POM, ROB, SHE, SZW, TAR, TOP, ZAN |
| Ortega-González et al. (2018) | Dogs | 0.93 | 2015 | Mexico | 10 | BRA, CAN, GRI, HAR, ICT, POM, POR, PYR, TAR, WOL |
| Ortega-Pacheco et al. (2020) | Cats | 0.89 |  | Mexico | 12 | AUS, AUT, BRA, CAN, GRI, HAR, ICT, PAN, PAT, POM, PYR, WOL |
| Perez-García et al. (2022) | Dogs | 0.86 | 2015 | Colombia | 9 | ALI, BRA, CAN, CAS, GRI, HAR, ICT, POM, TAR |
| Pinto-Ferreira et al. (2019) | Dogs | 0.89 | 2014 | Brazil | 10 | BRA, BUT, CAN, CAS, COP, GRI, HAR, ICT, POM, PYR |
| Pratt et al. (2017) | Dogs and cats | 0.93 | 2014 | Saint Kitts | 21 | ALE, AUS, AUT, BAL, BAT, BOR, BRA, CAN, CEL, CYN, DJA, GEO, GRI, HAR, ICT, JAV, MAN, POM, PYR, TAR, WOL |
| Ribeiro et al. (2021) | Cats | 0.96 | 2015 | Brazil | 20 | AUS, AUT, BRA, BUT, CAN, CANT, CAS, COP, DJA, GRI, HAR, HARB, HEB, ICT, POM, PYR, SEN, SHE, TAR, WOL |
| Rodríguez et al. (2014) | Cats | 0.96 | 2010 | Canada | 6 | BRA, CAN, GRI, HAR, ICT, POM |
| Said et al. (2018) | Dogs | 0.96 | 2016 | Tanzania | 6 | GRI, HEB, KEN, LOR, POM, SOK |
| Sant’Anna et al. (2017) | Dogs | 0.89 |  | Brazil | 8 | AUT, BAT, BRA, CAN, COP, GRI, ICT, POM |
| Scahill et al. (2022) | Dogs | 0.93 | 2019 | Sweden | 9 | AUT, BRA, CAN, COP, GRI, ICT, POM, SAX, SEJ |
| Seghesso Zabala et al. (2013) | Dogs | 0.68 |  | Argentina | 6 | CAN, CAS, GRI, ICT, POM, PYR |
| Sevá et al. (2020) | Dogs | 0.86 | 2015 | Brazil | 23 | AUS, AUT, BAT, BRA, BUT, CAN, CAS, COP, CYN, GRI, GUA, HAR, HARB, HEB, ICT, JAV, PAN, POM, PYR, SEN, SHE, TAR, WHI |
| Silva et al. (2017) | Dogs | 0.86 | 2010 | Brazil | 22 | AUS, AUT, BAT, BRA, BUT, CAN, CAS, COP, CYN, GRI, HAR, HEB, ICT, JAV, PAN, POM, PYR, SEN, SHE, TAR, WHI, WOL |
| Silva et al. (2018) | Dogs | 0.96 | 2014 | Brazil | 22 |  |
| Spangler et al. (2020) | Dogs and cats | 0.89 | 2017 | United States | 7 | AUT, BRA, CAN, GRI, HAR, ICT, POM |
| Sprißler et al. (2019) | Cats | 0.96 | 2016 | Thailand | 24 | ANH, AUS, AUT, BAL, BAT, BRA, BRO, CAN, CEL, COP, COX, CYN, DJA, GRI, HAE, ICT, KHO, PAI, PAT, POM, PYR, RAC, SAX, SEJ |
| Vojinovic et al. (2015) | Dogs | 0.86 | 2010 | Serbia | 8 | AUS, BAT, BRA, CAN, GRI, ICT, POM, SEJ |
| da Silva et al. (2016) | Dogs | 1.00 |  | Brazil | 25 | AND, AUS, AUT, BAT, BRA, BUT, CAN, CAS, COP, CYN, DJA, GRI, HAR, HEB, ICT, JAV, PAN, PAT, POM, PYR, SEN, SHE, TAR, WHI, WOL |
| da Silva et al. (2017) | Dogs | 1.00 | 2015 | Brazil | 24 | AND, AUS, AUT, BAT, BRA, BUT, CAN, CAS, COP, CYN, GRI, GUA, HAR, HARB, HEB, ICT, JAV, PAN, PAT, POM, PYR, SHE, TAR, WOL |
| de Abreu et al. (2019) | Dogs | 0.96 | 2013 | Brazil | 22 | AUS, AUT, BAT, BRA, BUT, CAN, CAS, COP, CYN, GRI, GUA, HAR, HEB, ICT, JAV, PAN, POM, PYR, SEN, SHE, TAR, WHI |
| de Lima Brasil et al. (2014) | Cats | 0.86 | 2011 | Brazil | 24 | AND, AUS, AUT, BAT, BRA, BUT, CAN, CAS, COP, CYN, GRI, HAR, HEB, ICT, JAV, PAN, PAT, POM, PYR, SEN, SHE, TAR, WHI, WOL |
| de Lima Brasil et al. (2018) | Dogs | 1.00 | 2015 | Brazil | 20 | AUS, AUT, BAT, BRA, CAN, CAS, COP, CYN, DJA, GRI, GUA, HAR, HARB, HEB, ICT, PAN, POM, SEJ, TAR, WOL |
| de Oliveira et al. (2016) | Dogs | 0.75 | 2014 | Brazil | 22 | AUS, AUT, BAT, BRA, BUT, CAN, CAS, COP, CYN, FOR, GRI, HAR, HEB, ICT, PAN, POM, PYR, SEN, SHE, TAR, WHI, WOL |
| de Paula Dreer et al. (2013) | Dogs | 0.93 | 2011 | Brazil | 22 |  |
| de Souza Rocha et al. (2022) | Dogs and cats | 0.69 | 2014 | Brazil | 19 | AND, AUS, AUT, BAT, CAN, CAS, CEL, CYN, DJA, GRI, HAR, HEB, ICT, PAN, PAT, POM, PYR, SHE, TAR |
| do Nascimento Benitez et al. (2021) | Dogs | 1.00 | 2015 | Brazil | 9 | BRA, BUT, CAN, CAS, COP, GRI, HAR, ICT, POM |
| do Nascimento Benitez et al. (2012) | Dogs | 1.00 | 2010 | Brazil | 23 | AUS, AUT, BAL, BAT, BRA, BUT, CAN, CAS, COP, CYN, FOR, GRI, HAR, HEB, ICT, PAN, POM, PYR, SEN, SHE, TAR, WHI, WOL |
| dos Santos et al. (2017) | Dogs and cats | 0.71 | 2013 | Brazil | 24 | AND, ARB, AUS, AUT, BAT, BRA, BUT, CAN, COP, CYN, GRI, GUA, HAR, HEB, ICT, JAV, PAN, PAT, POM, PYR, SHE, TAR, WHI, WOL |
| Žákovská et al. (2020) | Cats | 0.96 | 2013 | Czech Republic | 8 | BRA, CAN, GRI, ICT, POM, PYR, SEJ, SOR |
