# Supplementary material for: Seroprevalence of pathogenic Leptospira serogroups in asymptomatic domestic dogs and cats: systematic review and meta-analysis
Source: Front Vet Sci. 2024 Feb 16;11:1301959. doi: 10.3389/fvets.2024.1301959 (PMC10904519; doi:10.3389/fvets.2024.1301959)
Supplement: Supplementary file 2 [file Table_2.DOCX]

Supplementary material 2

**Seroprevalence of pathogenic *Leptospira* serogroups in asymptomatic dogs and cats: systematic review and meta-analysis**

Tamara Ricardo, Lucía Isabel Azócar-Aedo, María Andrea Previtali, Gustavo Monti*

*Correspondence: Gustavo Monti: [gustavo.monti@wur.nl](mailto:gustavo.monti@wur.nl)

## Supplementary Tables

Supplementary Table 2. Frequency of tested and detected serovars in dog studies and cat studies. SG: serogroup; SV: serovar; N cats: number of cat studies; N dogs: number of dog studies; N dogs & cats : number of dog and cat studies; Fr: frequency of detection (%) of the serovar among cat or dog effect estimates; k: number of effect estimates. The names of the serogroups were collapsed to their first three letters. UND: undetermined.

| SG | SV | N cats | N dogs | N dogs & cats | Fr. cats (k = 96) | Fr. dogs (k = 366) |
| --- | --- | --- | --- | --- | --- | --- |
| AND | Andamana | 2 | 8 | 2 | 1 (1.0%) | 0 (0.0%) |
| AUS | Australis | 14 | 36 | 5 | 5 (5.2%) | 13 (3.6%) |
|  | Bratislava | 14 | 36 | 4 | 6 (6.2%) | 16 (4.4%) |
|  | Lora | 1 | 1 | 0 | 0 (0.0%) | 1 (0.3%) |
|  | Muenchen | 1 | 1 | 0 | 0 (0.0%) | 0 (0.0%) |
| AUT | Alice | 0 | 1 | 0 | 0 (0.0%) | 1 (0.3%) |
|  | Autumnalis | 15 | 35 | 6 | 4 (4.2%) | 16 (4.4%) |
|  | Bim | 1 | 0 | 0 | 0 (0.0%) | 0 (0.0%) |
|  | Bulgarica | 0 | 1 | 0 | 0 (0.0%) | 0 (0.0%) |
|  | Butembo | 2 | 17 | 1 | 0 (0.0%) | 15 (4.1%) |
|  | Fortbragg | 0 | 5 | 0 | 0 (0.0%) | 1 (0.3%) |
|  | Rachmati | 4 | 0 | 0 | 1 (1.0%) | 0 (0.0%) |
| BAL | Arborea | 1 | 1 | 1 | 1 (1.0%) | 0 (0.0%) |
|  | Ballum | 8 | 11 | 2 | 7 (7.3%) | 4 (1.1%) |
|  | Castellonis | 6 | 25 | 2 | 3 (3.1%) | 7 (1.9%) |
|  | Kenya | 0 | 2 | 0 | 0 (0.0%) | 2 (0.5%) |
| BAT | Bataviae | 12 | 35 | 5 | 6 (6.2%) | 13 (3.6%) |
|  | Losbanos | 0 | 1 | 0 | 0 (0.0%) | 0 (0.0%) |
|  | Paidjan | 1 | 0 | 0 | 0 (0.0%) | 0 (0.0%) |
| CAN | Broomi | 1 | 0 | 0 | 0 (0.0%) | 0 (0.0%) |
|  | Canicola | 21 | 51 | 6 | 5 (5.2%) | 40 (10.9%) |
|  | Portlandvere | 0 | 1 | 0 | 0 (0.0%) | 1 (0.3%) |
| CEL | Anhoa | 1 | 0 | 0 | 0 (0.0%) | 0 (0.0%) |
|  | Celledoni | 5 | 6 | 4 | 1 (1.0%) | 3 (0.8%) |
|  | Whitcombi | 1 | 15 | 1 | 0 (0.0%) | 0 (0.0%) |
| CYN | Cynopteri | 10 | 26 | 5 | 3 (3.1%) | 9 (2.5%) |
| DJA | Djasiman | 4 | 11 | 4 | 3 (3.1%) | 7 (1.9%) |
|  | Sentot | 2 | 15 | 0 | 0 (0.0%) | 2 (0.5%) |
| GRI | Grippotyphosa | 20 | 51 | 6 | 8 (8.3%) | 32 (8.7%) |
|  | Ratnapura | 0 | 1 | 0 | 0 (0.0%) | 0 (0.0%) |
|  | Vanderhoedeni | 1 | 0 | 0 | 0 (0.0%) | 0 (0.0%) |
| HEB | Borincana | 0 | 1 | 1 | 0 (0.0%) | 0 (0.0%) |
|  | Hebdomadis | 7 | 32 | 4 | 2 (2.1%) | 4 (1.1%) |
|  | Kremastos | 1 | 1 | 0 | 0 (0.0%) | 0 (0.0%) |
| HUR | Hurstbridge | 0 | 1 | 0 | 0 (0.0%) | 0 (0.0%) |
| ICT | Copenhageni | 10 | 31 | 3 | 0 (0.0%) | 11 (3.0%) |
|  | Icterohaemorrhagiae | 20 | 50 | 6 | 8 (8.3%) | 32 (8.7%) |
|  | Lai | 2 | 5 | 1 | 0 (0.0%) | 2 (0.5%) |
|  | Mankarso | 0 | 1 | 1 | 0 (0.0%) | 0 (0.0%) |
|  | Sokoine | 0 | 2 | 0 | 0 (0.0%) | 2 (0.5%) |
| JAV | Coxi | 1 | 0 | 0 | 0 (0.0%) | 0 (0.0%) |
|  | Javanica | 7 | 17 | 4 | 6 (6.2%) | 6 (1.6%) |
|  | Poi | 2 | 1 | 0 | 1 (1.0%) | 1 (0.3%) |
|  | Sorexjalna | 1 | 0 | 0 | 0 (0.0%) | 0 (0.0%) |
| LOU | Louisiana | 0 | 1 | 1 | 1 (1.0%) | 2 (0.5%) |
| MAN | Lichuan | 0 | 1 | 0 | 0 (0.0%) | 0 (0.0%) |
| MIN | Georgia | 0 | 1 | 1 | 0 (0.0%) | 0 (0.0%) |
|  | Mini | 3 | 1 | 1 | 0 (0.0%) | 1 (0.3%) |
|  | Szwajizak | 0 | 1 | 0 | 0 (0.0%) | 0 (0.0%) |
| PAN | Panama | 6 | 23 | 3 | 0 (0.0%) | 3 (0.8%) |
| POM | Pomona | 20 | 51 | 6 | 11 (11.5%) | 26 (7.1%) |
|  | Proechimys | 1 | 0 | 0 | 0 (0.0%) | 0 (0.0%) |
| PYR | Alexi | 0 | 1 | 1 | 0 (0.0%) | 0 (0.0%) |
|  | Manilae | 0 | 1 | 0 | 0 (0.0%) | 1 (0.3%) |
|  | Pyrogenes | 16 | 34 | 5 | 3 (3.1%) | 21 (5.7%) |
|  | Robinsoni | 0 | 1 | 0 | 0 (0.0%) | 0 (0.0%) |
|  | Zanoni | 0 | 1 | 0 | 0 (0.0%) | 1 (0.3%) |
| RAN | Ranarum | 0 | 1 | 0 | 0 (0.0%) | 1 (0.3%) |
| SAR | Sarmin | 0 | 1 | 0 | 0 (0.0%) | 1 (0.3%) |
| SEJ | Guaricura | 0 | 8 | 1 | 0 (0.0%) | 3 (0.8%) |
|  | Haemolytica | 1 | 0 | 0 | 0 (0.0%) | 0 (0.0%) |
|  | Hardjo bovis | 4 | 8 | 2 | 0 (0.0%) | 2 (0.5%) |
|  | Hardjo prajitno | 17 | 37 | 5 | 0 (0.0%) | 13 (3.6%) |
|  | Medanensis | 0 | 1 | 0 | 0 (0.0%) | 0 (0.0%) |
|  | Saxkoebing | 3 | 2 | 0 | 0 (0.0%) | 0 (0.0%) |
|  | Sejroe | 6 | 9 | 0 | 3 (3.1%) | 4 (1.1%) |
|  | Wolffi | 5 | 20 | 3 | 0 (0.0%) | 4 (1.1%) |
| SEM | Patoc | 7 | 11 | 4 | 5 (5.2%) | 3 (0.8%) |
|  | Semaranga | 2 | 2 | 0 | 0 (0.0%) | 2 (0.5%) |
| SHE | Shermani | 4 | 22 | 3 | 1 (1.0%) | 7 (1.9%) |
| TAR | Malaysia | 2 | 2 | 1 | 0 (0.0%) | 0 (0.0%) |
|  | Tarassovi | 10 | 39 | 5 | 1 (1.0%) | 11 (3.0%) |
|  | Topaz | 0 | 1 | 0 | 0 (0.0%) | 1 (0.3%) |
|  | Vughia | 0 | 0 | 1 | 0 (0.0%) | 1 (0.3%) |
| UND | Cantagalo | 1 | 0 | 0 | 0 (0.0%) | 0 (0.0%) |
|  | Khorat | 1 | 0 | 0 | 0 (0.0%) | 0 (0.0%) |
